# Unveiling synergy in ultrasound-assisted enzymatic extraction: Role of treatment sequence and biomass complexity^☆^

**DOI:** 10.1016/j.ultsonch.2026.107749

**Published:** 2026-01-16

**Authors:** Bashar Kabawa, Imca Sampers, Katleen Raes

**Affiliations:** Research Unit VEG-i-TEC, Department of Food Technology, Safety and Health, Faculty of Bioscience Engineering, Ghent University, Campus Kortrijk, Graaf Karel de Goedelaan 46, 8500 Kortrijk, Belgium

**Keywords:** Ultrasound, Enzymatic assisted extraction, Pectin, By-products

## Abstract

Emerging extraction methods, such as enzyme-assisted extraction (EAE) and ultrasound-assisted extraction (UAE), are considered safer and more sustainable alternatives to conventional techniques, due to their reduced solvent usage. Nevertheless, their application remains limited due to their low efficiency and sensitivity to environmental conditions. To overcome these drawbacks, ultrasound-assisted enzymatic extraction (UAEE) has been proposed as an alternative synergistic approach to improve biomass disruption. In this study, the effect of ultrasound on the susceptibility of pectic substrates to enzymatic degradation was evaluated using both purified pectin and pectin-rich complex matrices (grapefruit peels and apple pomace). The extent of enzymatic degradation was assessed by monitoring the release of reducing sugars, while microscopic evaluation of the cell microstructure, the total phenolic release (TPC) and metal element release were quantified to support the findings.

Results indicate that ultrasonic pre- and post-treatments had no significant impact on pectin hydrolysis. In contrast, when ultrasound was applied during the enzymatic reaction, the extent of hydrolysis increased, but only in the case of the complex matrices, indicating a synergistic effect. The increased release of calcium and potassium ions suggested that ultrasound induced an enzymatic cofactor release from biomass, contributing to improved enzyme activity. Similar degradation of the cell microstructure was observed in the case of ultrasonic post treatment, although without improved hydrolysis of cell-wall pectin. This implies that prior enzymatic action weakened the matrix, making it more fragile. This is the first study to investigate the impact of plant matrix structure on the synergistic effect of the ultrasound-enzyme combination.

## Highlights

• The impact of ultrasound on the susceptibility of different pectic substrates to enzymatic degradation was evaluated.
• The effect of substrate complexity was assessed using both purified pectin and pectin-rich biomasses.
• Ultrasound enhanced enzymatic degradation only in complex biomass.
• The coupling mode of ultrasonic and enzymatic treatments strongly affected the enzymatic reaction.
• Simultaneous ultrasonic and enzymatic treatment promoted enzymatic cofactor release, improving enzyme activity.

## Graphical abstract

**Figure:**
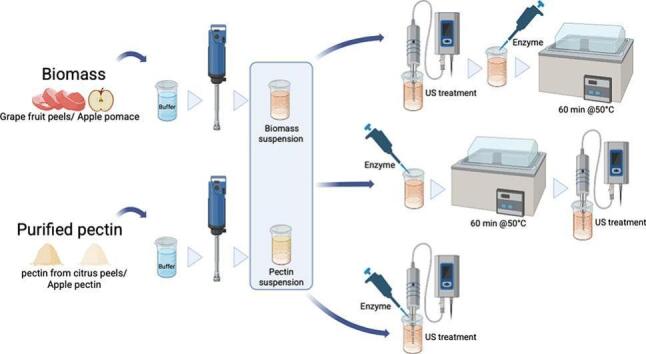

## 1 Introduction

The fruit and vegetable processing industry is increasingly shifting toward ready-to-eat and convenient products to meet changing consumer lifestyles, while advancements in processing technology, seasonal crop management, and expanding export opportunities continue to shape its growth. However, this growth has been accompanied by an increase in agro-industrial side streams, including waste and by-products. Agro-industrial by-products might include both inedible components, such as stems, peels, pits, leaves, straws, and pomaces, as well as some edible parts that fail to meet the production standards, such as shape, size, or color [1]. These by-products, however, are often rich in nutrients, functional components, and bioactive compounds, such as proteins, peptides, polysaccharides, dietary fibers, and phenolic compounds [1], [2]. The disposal of this biomass not only poses a major challenge due to the associated impact on the environment. It is also considered a waste of scarce resources that, if properly valorized, can lead to the development of value-added products [3]. A major step in the valorization of agro-industrial by-products is the extraction of these valuable compounds, thereby enabling their further utilization in various applications [4].

Several conventional and emerging extraction techniques are currently available and could potentially be utilized for this purpose. The fundamental principle of all these methods is based on two consecutive mechanisms: first, the disruption of the cell wall of biomass cells, followed by the extraction of valuable components from the intracellular medium using the extracting power of different solvents [5], [6]. Ultrasound-assisted extraction (UAE) and enzyme-assisted extraction (EAE) are two examples of emerging techniques frequently used for this purpose. The common advantages of such emerging methods over conventional ones are their reduced usage of chemical solvents, as well as lower energy consumption and shorter extraction times [7].

In ultrasound-assisted extraction, the energy of ultrasonic waves is utilized to degrade cell walls and promote the release of intracellular components. Ultrasound is defined as sound waves at frequencies beyond the detection range of the human ear [8]. Due to the physical nature of sound as a state of alternating compression and rarefaction cycles, it creates a local displacement of the medium molecules through which it propagates. When this medium is incompressible, as in liquids, ultrasonic frequencies can generate rarefaction zones strong enough to displace liquid molecules beyond their critical molecular distance, resulting in the formation of microbubbles, referred to as cavities [9]. These cavities then, in the case of low ultrasonic frequencies (20–100 kHz), implode rapidly, producing free radicals, vortices, localized heat, and intense shear forces. Collectively, these effects disrupt the cell walls of the biomass cells located near these bubbles, thereby facilitating the extraction of intracellular components [10], [11].

On the other hand, in enzyme-assisted extraction (EAE), hydrolyzing enzymes are utilized to selectively target specific cell-wall constituents, thereby leading to the disruption and degradation of the cellular wall of the treated biomass and enabling efficient extraction of the valuable components [12]. The primary advantages of EAE over conventional extraction methods lie in its non-thermal and aqueous-based nature, making it more suitable for extracting sensitive and thermolabile compounds. Additionally, EAE enables the extraction of not only free intracellular compounds but also those bound to the cell wall, which are typically unextractable through conventional techniques [6]. However, the high cost and the high sensitivity to the environment are the two major limitations of EAE for large-scale applications [6], [13].

To overcome these obstacles, ultrasound-assisted enzymatic extraction (UAEE) has recently been proposed as a hybrid technique that combines ultrasonic treatment with enzyme-assisted extraction. Numerous studies have reported the positive effects of ultrasonic (US) treatment on enzymatic reactions, and various theories have been suggested to explain these observed enhancements. However, there is still no complete understanding or consensus on a specific mechanism underlying the observed enhancement. Some studies suggested a direct effect of US treatment on enzymes, represented as an increase in enzyme activity due to sonication under mild conditions [14], [15], [16], [17], [18]. In contrast, other studies attributed this effect to the impact of US treatment on the substrate itself. Under specific process parameters, US treatment can modify the substrate structure, making it more accessible to the active sites of the enzyme, remove any protective layer limiting the enzymatic hydrolysis, or even partially disintegrate and depolymerize the treated substrate [19], [20], [21]. Additionally, some studies highlighted the mixing and agitation phenomena caused by ultrasound-induced cavitation and their role in improving mass transfer, as a possible reason for the enhancement in hydrolysis efficiency upon sonication [17], [22], [23].

In a previous study, the effect of US treatment on enzyme activity was investigated [24]. It was established that US treatment does not necessarily affect enzyme activity, although it may alter enzyme conformation. Building on this, the current work aims to investigate the effect of US treatment on the susceptibility of substrates to enzymatic degradation. For this purpose, both purified pectic substrate and pectin-rich complex matrix containing the same type of pectin were subjected to US treatment, before, during, and after enzymatic hydrolysis. To the best of our knowledge, this is the first study to assess the influence of a combined US and enzymatic treatment on both complex and purified pectic substrates. The choice of pectic substrates was based on the widespread occurrence of pectin as a key cell wall component in various fruit and vegetable by-products [25], [26].

## 2 Materials and methods

### 2.1 Materials

Purified pectin from citrus peel (PCP) and purified pectin from apple (PAP), with degrees of methyl esterification of 52% and 68% respectively, were obtained from Sigma Aldrich (Darmstadt, Germany). Pectinase from *Aspergillus aculeatus* (Pectinex® Ultra SP-L, a blend of pectinases, hemicellulases and β-glucanases with activity of 3300 PGNU/g), 3,5-dinitro salicylic acid, Folin Ciocalteu′s phenol reagent, gallic acid, and D-(+)-galacturonic acid monohydrate were also purchased from Sigma Aldrich (Darmstadt, Germany). Citric acid, sodium hydroxide, sodium carbonate, and L-(+)-potassium sodium tartrate tetrahydrate were purchased from VWR Chemicals (Leuven, Belgium), while di-sodium hydrogen phosphate anhydrous was obtained from Chem-Lab (Zedelgem, Belgium). Fresh apple (cv. Jonagold) and fresh red grapefruit (cv. Star Ruby) were obtained from a local supermarket (Kortrijk, Belgium).

### 2.2 Sample preparation

Fresh apples (3 kg) were washed, cut into pieces approximately 5 cm in size, and deseeded. These pieces were then juiced using a slow juicer (AG-8500 s by Angel, Busan, Korea) and the resulting pomace was collected. The pomace (POM) was then washed with 2 L of demineralized water for 5 min, drained and reintroduced into the juicer to remove excess washing water.

Grapefruit (7 kg) were manually peeled by removing both the albedo and flavedo from the fruit. The peels were cut into pieces of around 5 cm, gently rinsed under running demineralized water, and ground in a knife mill (Grindomix GM200, Retsch GmbH, Haan, Germany) at 4000 rpm for 30 s in intermittent mode. The grapefruit peels (GFP) were then divided into sample cups, 80 g each, and washed twice by shaking with 350 mL distilled water at 200 rpm for 60 min using an orbital shaker (Stuart SSL1, by Cole-Parmer, Stone, Staffordshire, United Kingdom). The washing water was drained using cheesecloth after each step. The prepared fruit by-products were then stored in portions at −40 °C until further use.

Samples of commercial purified pectin suspension (PCP and PAP; 7.5 g/L) and fruit by-product suspension (POM and GFP; 40 g/L), resulting in about 1 g/L pectin as estimated based on literature values [27], [28], [29], [30], [31], [32], [33], were prepared by dispersing the appropriate amount of the respective substrate in 150 mL or 120 mL of 10 mM citrate buffer (pH 4.8), depending on the type of treatment. Samples of 150 mL were sonicated either before or after enzymatic hydrolysis. In comparison, the 120 mL samples were sonicated directly after mixing with 30 mL of enzyme solution, to maintain both the sample volume and the substrate-enzyme ratio used in the pre- and post-enzymatic hydrolysis of sonicated substrate suspension. The concentration of the by-product was selected at the highest possible concentration at which the suspension still retained a liquid consistency. The suspensions were then homogenized using an ultraturrax (T18 digital by IKA, Staufen, Germany) at 16000 rpm for 15 sec.

### 2.3 Equipment and ultrasonic treatment

All US treatments in this study were carried out using a probe-type ultrasonic device (Model UP200st by Hielscher, Germany) with a driven frequency of 26 kHz, equipped with a 14 mm diameter (154 mm^2^ surface) probe. Suspensions were directly sonicated by immersing the tip of the ultrasonic horn in the suspension at a depth of 1 cm. All US treatments were performed at 140 W with a duty cycle of 50% (10 s on and 10 s off) and a controlled treatment temperature between 48 °C and 52 °C by using a cooled water bath. The total net sonotrode output US energy was monitored in all executed US treatments.

### 2.4 Experimental setup

#### 2.4.1 Ultrasonic pretreatment followed by the enzymatic hydrolysis

Different substrate suspensions (150 mL) were first sonicated for 60 min, during which the net output US energy ranged between 225,060 and 243,900 J, which corresponds to 200,053 and 216,800 J/g pectin in the case of purified pectin. Afterward, enzymatic hydrolysis was initiated by adding 1 mL of 0.1% (w/v) pectinase solution prepared in citrate buffer to 4 mL of the pretreated substrate suspension. This addition resulted in a final enzyme concentration of 0.02% (w/v) in the reaction mixture, corresponding to an enzyme dosage of 110 PGNU/g pectin in the case of purified pectin. Hydrolysis was conducted at 50 °C using a warm water bath for various incubation times (0, 5, 10, 15, 30, 60 min). At the end of the incubation time, the reaction was terminated by adding 180 µl of NaOH (1 M) to elevate the pH of the reaction mixture beyond the enzyme's active range and samples were immersed in an ice bath until analysis. A control hydrolysis was carried out using the same substrate with no previous US pretreatment.

#### 2.4.2 Ultrasonic treatment during enzymatic hydrolysis

Different substrate suspensions (120 mL) were preincubated in a water bath until the temperature reached 50 °C. Then, 30 mL of 0.1% (w/v) pectinase solution in citrate buffer, resulting in 0.02% (w/v) pectinase in the final mixture, was added, manually mixed with the substrate, and immediately subjected to US treatment. Aliquots of 2 mL were taken at specific time slots and transferred to tubes prefilled with 72 µl NaOH (1 M) and kept in an ice bath till further analysis. A control hydrolysis was performed following the same setup in a warm water bath without applying ultrasound.

#### 2.4.3 Ultrasonic treatment after the enzymatic hydrolysis

Fruit by-product samples (120 mL) were preheated in a water bath until the temperature reached 50 °C. Then, 30 mL of 0.1% (w/v) pectinase solution in citrate buffer, resulting in 0.02% (w/v) pectinase in the final mixture, was added, mixed well, and incubated at 50 °C for 60 min. At the end of the incubation time, the enzymatic hydrolysis was terminated by adding 5.4 mL of 1 M NaOH, followed by immediate cooling in an ice bath. This mixture was then sonicated for 60 min, using the process parameters described in section 2.3 except for the sonication temperature. To avoid any possible reactivation of the enzyme during this step, the samples were kept in an ice bath throughout the US treatment. Since some temperature increase during sonication was unavoidable, the temperature of the mixture was continuously monitored, and the maximum recorded value was 35 °C. For comparison, the same procedure was conducted without the final US treatment and used as a control.

### 2.5 Analytical assays

#### 2.5.1 Susceptibility to enzymatic degradation

The susceptibility of pectic substrates to enzymatic degradation was assessed by quantifying the released reducing groups resulting from the enzymatic hydrolysis of each respective sample at specific predetermined time slots. The concentration was determined using the di-nitrosalicylic acid method (DNS) as proposed by Miller (1959), with minor modifications [34]. Briefly, 4 mL of DNS reagent was added to 1 mL of the reaction aliquot, and the mixture was incubated in a boiling water bath for 10 min. The samples were then cooled to room temperature by immersing them in a cooled water bath, and the absorbance was measured at 550 nm using a UV–VIS spectrophotometer (UV-1800, Shimadzu, Duisburg, Germany). Concentrations were calculated using external standard curves of D-(+)-galacturonic acid monohydrate. The limits of detection (LOD) and quantification (LOQ) were maintained below 0.19 and 0.57 mg/mL, respectively.

#### 2.5.2 Microscopic analysis

The microstructure of GFP treated by ultrasound and/or enzyme was assessed using light microscopy. The samples were centrifuged in 50 mL Falcon tubes at 4000 rpm for 10 min, and the sediments were suspended afterward in deionized water on a microscope slide. Samples were analyzed using a Nikon Eclipse Ti microscope (Nikon Corporation, Tokyo, Japan), and images were acquired at 400× magnification using an Andor DU-885 scientific CCD camera (Oxford Instruments, Belfast, UK).

#### 2.5.3 ICP-OES analysis

The content of different elements (Ca, Na, Mg, Fe, and K) released from GFP samples subjected to various treatments was determined using an inductively coupled plasma optical emission spectrometer (ICP-OES; Thermo Fisher Scientific, iCAP 7000 Series). Briefly, 50 mL of each treated sample was transferred into 50 mL centrifuge tubes and centrifuged at 4000 rpm for 15 min at 4 °C. The resulting supernatant was collected and digested using a microwave digestion system (Anton Paar Multiwave 5000) in the presence of concentrated nitric acid (HNO_3_). After digestion, the solutions were diluted with double-distilled water to a defined final volume. Quantification was performed using a multi-element calibration standard ranging from 0.2 to 20 ppm. The LOD and LOQ corresponding to the standard curve of each element are indicated in Table 1. The instrument was operated under standard, manufacturer-recommended conditions, with element-specific wavelengths selected for detection.

**Table 1:** Limit of detection (LOD) and limit of quantification (LOQ) for the quantified element by the ICP-OES analysis.

Element	LOD (ppm)	LOQ (ppm)
Ca	0.83	2.52
Mg	0.64	1.94
Fe	0.77	2.34
Na	0.73	2.22
K	0.73	2.20

#### 2.5.4 Assessment of the total phenolic content (TPC) of the fruit by-product

The total phenolic content (TPC) was determined using Folin-Ciocalteu (FC) reagent as described by Singleton et al. (1999) [35]. Briefly, 1 mL sample/standard was mixed with 1 mL distilled water and 0.5 mL FC reagent (10 times diluted) and then incubated in the dark for 6 min. Then, 1.5 mL of sodium carbonate solution (20% v/v) was added and incubated in the dark at room temperature for 120 min. The absorbance was measured at 760 nm using a UV–VIS spectrophotometer (UV-1800, Shimadzu, Duisburg, Germany). TPC was expressed as mg/L gallic acid equivalent. The LOD and LOQ were 1.48 mg/L and 4.48 mg/L, respectively.

### 2.6 Statistical analysis

All experiments were conducted in triplicate, and values are reported as mean ± standard deviation (SD). Statistical analysis was performed using SPSS statistical software version 29.0 (SPSS Inc., Chicago, IL, USA). One-way analysis of variance (ANOVA) with Welch’s correction was applied to compare the mean values of the element release and total phenolic content under different treatments, followed by Tukey’s post-hoc test and the Games-Howell test for pairwise comparisons. A student’s *t*-test was also performed to detect any significant increase in reducing sugar release by different treatments. The significant difference was accepted at p < 0.05.

## 3 Results and discussions

### 3.1 Effect of ultrasound on the enzymatic degradation of pectic substrates

**Fig. 1:**
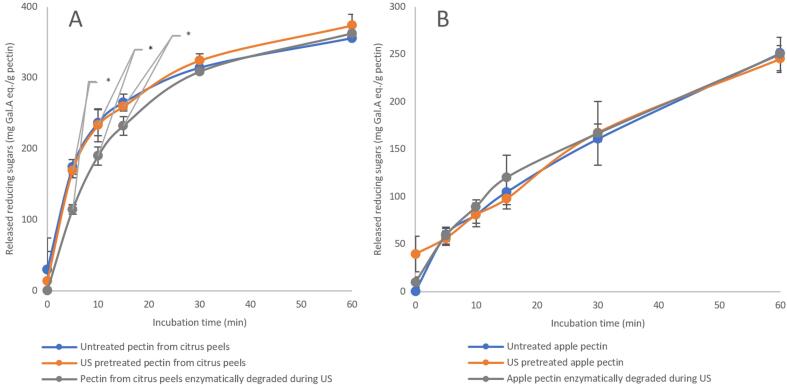
Susceptibility of purified pectin from (A) citrus peels; and (B) apple to enzymatic degradation due to 60 min of ultrasonic treatment applied prior to and during the enzymatic hydrolysis (n = 3). Points marked with a star exhibit significant differences.

To assess the impact of US treatment on the tendency of purified pectic substrates to enzymatic degradation, PCP and PAP samples were subjected to US treatments under defined process parameters. These treatments were applied either before or during pectinase hydrolysis, which, in turn, was conducted under optimal temperature and pH conditions. Fig. 1 presents a comparison of the released reducing sugars resulting from enzymatic hydrolysis of PCP and PAP in the presence and absence of US treatments applied in different modes. As shown, no significant change in the amount of released reducing sugars was observed upon US pretreatment for either pectin type, indicating that neither sample exhibited a change in their susceptibility to enzymatic degradation upon US pretreatment. This suggests that ultrasound did not lead to a change in pectin structure that can affect its enzymatic degradation rate. These results contradict previous reports in the literature, which indicate that US treatment induces multiple modifications in pectin. For instance, Ma et al. (2018) reported that US pretreatment induced an enhancement in the degree of enzymatic hydrolysis of citrus pectin, possibly due to the decrease in its molecular weight [36]. Similarly, Larsen et al. (2021) observed a decline in the average molecular weight of sugar beet pectin from 300 to 150 kDa, with a narrower molecular weight distribution, indicating improved homogeneity due to US treatment [37]. A change in rheological properties of sugar beet pectin (20 g/L) was also reported due to US treatment, including a decrease in intrinsic viscosity and viscosity-average molecular weight [38]. However, the used concentration was much higher than in our study (7.5 g/L), making the pectin viscosity in our research too low to be measured. An ultrasound-induced alteration of the secondary bonds of pectin chains has also been reported. This alteration leads to the formation of more flexible chains and coils, while partially disrupting the native pectin network in aqueous solutions [39]. Furthermore, Xu et al. (2023) found that ultrasound can break some glycosidic bonds in sugar beet pectin [40].

When ultrasound was applied to purified pectin solutions during enzymatic hydrolysis, a similar trend was observed, with some distinctions. As shown in Fig. 1, conducting the hydrolysis of PAP under US conditions did not result in a significant difference in the released reducing sugars compared to hydrolysis without ultrasound. However, when PCP was enzymatically hydrolyzed under US conditions, a significant decrease in the released reducing sugars was observed during the initial phase of hydrolysis, up to 15 min of incubation (p = 0.008, 0.020, and 0.045 for 5, 10, and 15 min, respectively). This decrease was then compensated in later time slots, ultimately eliminating the significant differences between the US and non-US treated samples. In contrast, Ma et al. (2016) reported an enhancement in the enzymatic degradation of pectin when applied under US conditions. This increase was attributed to an induced increase in enzyme activity [41]. Larsen et al. (2021) reported an increase in the low MW fraction of the hydrolyzed pectin under US conditions as compared to that hydrolyzed in the absence of US. It was suggested that US treatment contributes to degrading the pectin into medium MW oligomers, while the enzyme degrades oligomers further into a lower MW fraction [37]. This enhancing effect of the ultrasound, simultaneously with enzymatic hydrolysis, was also seen for other polymers, e.g. chitin, although it should be taken into account that every polymer has different characteristics (such as water solubility and crystallinity). For chitin, it was also found that the hydrolysis was more extensive when the enzymatic degradation occurred under US conditions. This enhancement was mainly attributed to an intensified molecular collision between the chitin and the enzyme due to enhanced mass transfer induced by ultrasound. Furthermore, a breakage in glycosidic bonds due to ultrasound may also have contributed to this enhancement, as viscosity-average molecular weight was noticed to be decreased in this case as well [42]. However, the contradiction between the results of this study and previously reported findings is not unexpected. The US process parameters used in earlier studies differ strongly from those applied in the present work. For instance, in the study of Ma et al. [36], the US power density ranged from 9 to 27 W.mL^−1^, with the highest degree of enzymatic hydrolysis observed at 18 W.mL^−1^, whereas the power density used in our experiment was around 0.93 W.mL^−1^. Similarly, Larsen et al. [37], not only employed a continuous circulation system but also applied considerably higher US power levels (190 and 300 W in a 9 mL cell) compared to this study. Additionally, it is well known that reproducing ultrasound-related experiments is highly challenging due to several factors. These include the frequent lack of detailed methodological information, inconsistency in reporting US process parameters, and differences in the design and properties of different ultrasonic processors.

**Fig. 2:**
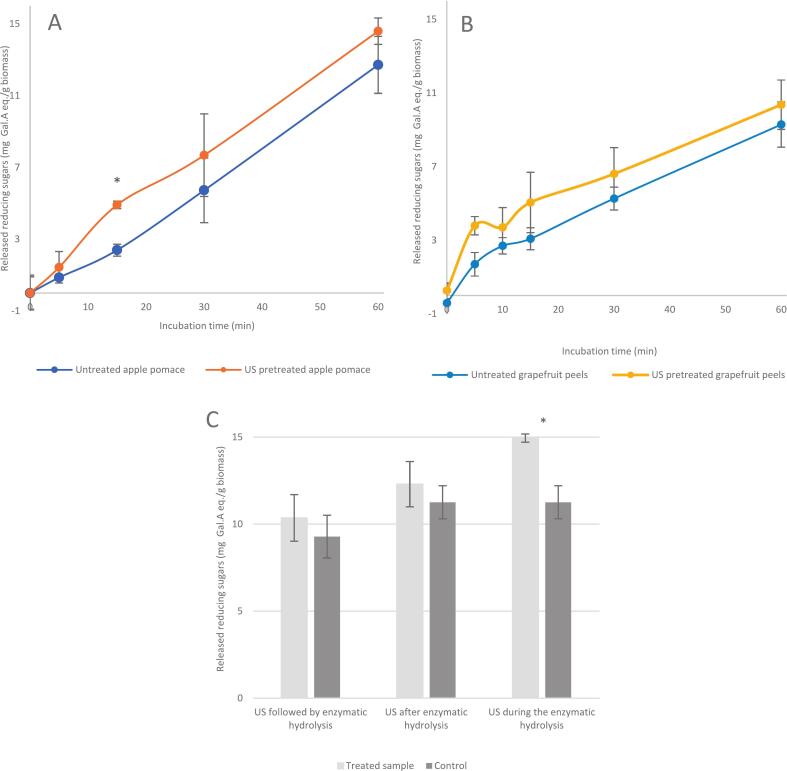
Effect of US treatment on the susceptibility of (A) apple pomace and (B) grapefruit peels to enzymatic degradation when ultrasound was applied prior to hydrolysis. Panel (C) shows a comparison of the released reducing sugars after 60 min of enzymatic degradation of grapefruit peels, when ultrasound was applied prior to, during, or after the hydrolysis (n = 3). Time slots and/or treatments marked with a star exhibit significant difference across treatments.

On the other hand, the number of reports addressing ultrasound-assisted enzymatic degradation of purified substrates, such as those discussed above, is minimal. Most of the available literature instead focuses on the application of ultrasound-enzyme conjugation with different biomass, whose matrix might have an impact on the observed effect. To investigate the effect of a complex matrix on the ultrasound-enzyme combination, grapefruit peels (GFP) and apple pomace (POM) were used as pectin-rich substrates, which offer a biomass complex matrix in addition to the same pectin structure used in the previous experiments. Fig. 2 illustrates the effect of US treatment on the susceptibility of these substrates for enzymatic degradation. As seen in Fig. 2, US pretreatment of both biomasses did not lead to any significant change in the amount of the released reducing sugars by an enzymatic hydrolysis. These results agree with those obtained from the previous experiments on purified pectin. In contrast, Lieu et al. (2010) observed a positive effect of US pretreatment on the juice recovery from grape mash. Indeed, ultrasound as a pretreatment contributed to degrading the cell walls, while the following pectinase application helped to degrade the released polysaccharides by the US treatment, thereby improving free-run juice recovery [43]. US pretreatment was also found to enhance the enzyme-assisted extraction of pectin from sugar beet pulp using both cellulase and xylanase [44]. This enhancement was attributed to the increased porosity resulting from the collapse of cavitation bubbles. This, in turn, improved the penetration of the enzymes into the plant tissues. Similarly, the enzymatic degradation of sugar cane bagasse cellulose showed a 21.3% improvement due to a prior US pretreatment [45]. This improvement increased proportionally with sonication time, indicating that the improvement in enzymatic hydrolysis was due to the mechanical forces induced by US cavitation. However, to the best of our knowledge, no studies have reported an increase in extraction yield resulting from the direct depolymerization of pectin by ultrasound. Existing reports on the degradative effects of ultrasound primarily focus on crystalline polysaccharides, whereas pectin is an amorphous polymer. This structural difference may explain the absence of a positive impact in our study. Previous research has shown that US treatment reduces the crystallinity and degree of polymerization of cellulose [46], but such effects may not apply to amorphous pectin.

However, when the enzymatic hydrolysis of pectin within these biomasses was conducted during US treatment, a significant increase in the release of reducing sugars was observed after 60 min, as compared to the control hydrolysis in the absence of ultrasound, as shown in Fig. 2 (C). These results suggest that ultrasound exhibits a synergistic effect with the enzymatic hydrolysis of pectin only when it is present in complex matrices. This synergistic effect might be explained as ultrasound can only degrade the plant cell wall when the latter has been weakened by the initial activity of pectinase. The observations from the microscopy analysis, as discussed in Section 3.2, further support this explanation. However, this hypothesis cannot explain the observed enhancement in the reducing sugar release during the simultaneous treatment of US and the enzymatic hydrolysis. Notably, when US treatment was carried out after enzymatic hydrolysis, no significant increase in the released reducing sugars was observed compared to the control. This suggests that the synergistic effect is not merely due to cell wall weakening by enzymes accompanied by ultrasound-induced disruption. Instead, an additional factor may be involved. It is possible that carrying out enzymatic hydrolysis under ultrasound conditions triggers the release of specific components from the biomass matrix, such as cofactors, that could enhance enzyme activity. Another possible explanation is the improved mass transfer due to the vortices formed in the reaction medium by the ultrasound-induced cavitation. An enhancement in protein extraction yield and total phenolic content from sesame bran was observed when ultrasound was applied in combination with enzymatic extraction. This synergistic effect was attributed to a possible enhancement of the enzyme-substrate interaction due to the US waves [47]. A similar observation was made when ultrasound was applied in combination with the enzymatic hydrolysis of newspaper waste. A 2.4-fold increase in the released reducing sugar for UAE hydrolysis was obtained as compared to the same hydrolysis obtained in the absence of ultrasound. It was concluded that ultrasound lowers the diffusion-limiting barrier to enzyme-substrate binding, thereby leading to an increase in the reaction rate [48].

### 3.2 The impact of different ultrasonic-enzymatic treatments on the microstructure of biomass cells

**Fig. 3:**
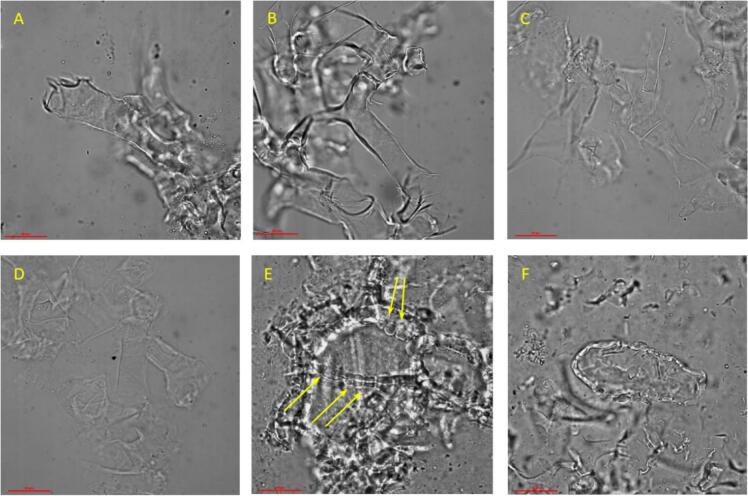
Light microscopy images of grapefruit peel cells at 400× magnification showing (A) Control sample with no previous treatment; (B) sample treated with ultrasound alone; (C) sample enzymatically degraded; (D) sample pretreated with ultrasound followed by enzymatic hydrolysis; (E) sample enzymatically degraded under US conditions; (F) sample treated with enzyme followed by ultrasound. Scale bar represents 50 µm and yellow arrows indicate pores and perforations in the cell walls.

To gain further insight into the mechanism governing the degradation of biomass cells by different treatments, microscopic visualization of grapefruit peel (GFP) samples was conducted after each treatment. As shown in Fig. 3 B, the cell wall structure of the grapefruit peel remained intact after 60 min of US treatment alone. A similar observation was also made after 60 min of pectinase hydrolysis alone (Fig. 3 C). While overall cell integrity was preserved in both cases, a slight alteration in the thickness of the cell walls after enzymatic hydrolysis was noticeable as compared to US-treated sample. This suggests a partial degradation of the cell wall pectin due to the enzymatic activity. This partial degradation likely weakened the cell walls without fully destroying their overall integrity. When enzymatic hydrolysis was applied to US-pretreated samples, no noticeable differences were observed compared to samples subjected to enzymatic treatment alone. In both cases, the integrity of the cell wall was not affected, as seen in Fig. 3 C and D, while cell walls became pale under the microscope, reflecting a deterioration in the cell wall thickness. This indicates that the US pretreatment applied prior to the enzymatic hydrolysis was insufficient to cause detectable alterations in the cell wall structure or to enhance enzymatic accessibility.

In contrast, when enzymatic hydrolysis was performed simultaneously with US treatment, the cell walls appeared to be completely disrupted and no intact structures were observed. Instead, clear pores and perforations were visible across the remaining distinguished cell walls. Additionally, fragments of cell wall debris were scattered throughout the field of view, indicating extensive structural breakdown (Fig. 3 E). These observations support the previously discussed results (Fig. 2 B), which showed that the simultaneous ultrasound-enzyme treatment led to a higher yield of reducing sugars compared to other treatments. This effect may be attributed to the combined action of both treatments. While ultrasound alone was not strong enough to disrupt intact cell walls, enzymatic hydrolysis initiated a partial degradation, thereby weakening the structural integrity of the cell walls and making them more susceptible to US cavitation, ultimately leading to complete disintegration. A similar effect was observed when US treatment was applied after enzymatic degradation, which further supports our hypothesis. To the best of our knowledge, no previous studies have visualized the impact of pectinase hydrolysis of biomass under US conditions.

### 3.3 Elemental analysis of ion releases due to different treatments

As previously suggested in this study, the synergistic effect produced by the simultaneous application of ultrasound and pectinase might be partially attributed to the initial enzymatic activity. According to this hypothesis, ultrasound becomes effective only after an initial action of pectinase. Only at this point, the cell walls become fragile enough to collapse under the impact of acoustic cavitation. However, this explanation, although supported by microscopic evidence, does not clarify why enhanced degradation of cell-wall pectin occurred only during the simultaneous ultrasound-enzyme treatment. In the sequential mode, where ultrasound was applied after enzymatic incubation, such enhancement was absent even though enzymatic weakening of the cells had already taken place.

Although both treatments showed comparable effects on the microstructure of the plant cell wall, only the simultaneous treatment further promoted enzymatic degradation of cell-wall pectin. This suggests that an additional factor unique to the simultaneous treatment contributed to the observed synergistic effect. To investigate this hypothesis, the content of various metal elements released into the aqueous phase in grapefruit peels suspension subjected to different treatments was quantified. As shown in Table 2, no significant increase in the release of metal ions upon US treatment alone was observed as compared with the control. This result is consistent with the microscopic observation of the cell wall, which confirmed that ultrasound alone has no impact on cell wall integrity. In contrast, a slight, but not significant, increase in the concentrations of Mg and Na was observed upon enzymatic treatment alone, while Ca and K significantly increased up to 2.12 and 2.92 mg/g GFP as compared to 1.49 and 2.05 mg/g GFP in the control, respectively. However, the simultaneous ultrasound-enzyme treatment led to a significant increase in the concentrations of all studied metal elements except Fe as compared with the control. The concentrations reached 0.43 and 44.4 mg/g GFP for Mg and Na, respectively, whereas the increase in Caand K reached 2.51 and 3.15 mg/g GFP, which were even higher than those observed under enzymatic treatment alone.

**Table 2:** The content of several elements in the aqueous fraction of grapefruit peel suspension after different treatments.

Type treatment	Ca (mg/g GFP)	Mg (mg/g GFP)	Fe (mg/g GFP)	Na (mg/g GFP)	K (mg/g GFP)
Control	1.49 ± 0.13a	0.32 ± 0.02a	0.18 ± 0.01a	42.91 ± 0.40a	2.05 ± 0.09a
Ultrasonic	1.69 ± 0.03a	0.38 ± 0.06a,b	0.22 ± 0.05a	43.44 ± 0.81a,b	2.10 ± 0.02a
Enzymatic	2.12 ± 0.01b	0.37 ± 0.01a,b	0.20 ± 0.04a	43.97 ± 0.14a,b	2.92 ± 0.04b
Ultrasound-Enzyme combined	2.51 ± 0.09c	0.43 ± 0.02b	0.18 ± 0.00a	44.40 ± 0.59b	3.15 ± 0.09c

These increases in metal ion release observed in the case of simultaneous ultrasound-enzyme treatment may explain the enhanced release of reducing sugars associated with this process. Enzymatic activity is well known to be strongly influenced by the presence of certain mineral ions, which can act as cofactors. Reports have indicated that the presence of Zn^2+^, Mg^2+^, Ca^2+^, Ba^2+^, Mn^2+^, and K^+^ can stimulate pectinase activity [49]. However, the term (pectinase) refers to a broad group of enzymes, which are often present as mixtures in commercially available preparations. Consequently, some minerals may enhance the activity of one pectinolytic enzyme while having no effect or inhibiting another, leading ultimately to either enhancement or inhibition in the enzymatic degradation of pectin. Pedrolli et al. [50] established that polygalacturonase is activated by Mg^2+^, Na^+^, and Mn^2+^ but inhibited by Ca^2+^ and Zn^2+^. Whereas in another study, both Ca^2+^ and Fe^2+^ were reported to have a positive effect on the same enzyme [51], [52]. Likewise, polygalacturonate lyase has been reported to be stimulated by Ca^2+^ and Mg^2+^ but inhibited by Cu^2+^, Fe^2+^, and Zn^2+^ [53]. Therefore, the overall effect of metal ions on a specific enzyme preparation depends on the composition and the origin of this preparation.

### 3.4 Assessment of the total phenolic compound yield

To evaluate the studied treatments as valid extraction techniques, the total phenolic content (TPC) released from the grapefruit peel suspensions after each treatment was quantified and used as an indicator for the efficiency of this extraction method. In plant matrices, phenolic compounds are typically present in both free and bound forms. Therefore, an efficient extraction technique should enhance the breakdown of the plant matrix and promote the release of bound phenolics.

**Fig. 4:**
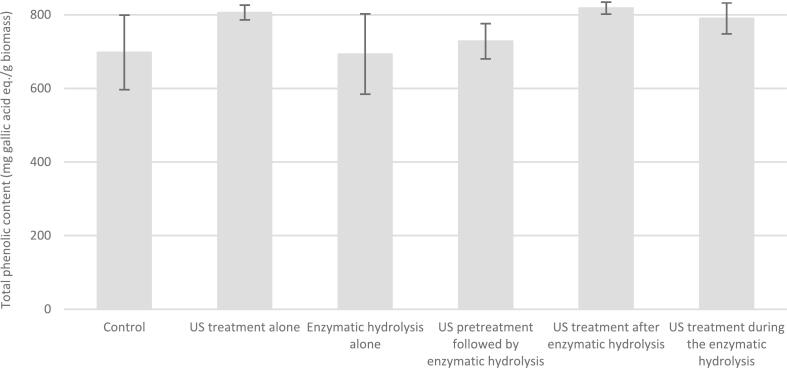
Total phenolic content of grapefruit peels suspension after different treatments of ultrasound and/or enzymatic hydrolysis. An untreated sample was chosen to be the control. (n = 3).

Although the simultaneous application of ultrasound during enzymatic incubation improved the enzymatic degradation of pectic substrates in biomass, no significant differences in total phenolic release were detected among the treatments (Fig. 4). This result might be attributed to the fact that pectin is not the main barrier retaining phenolic compounds within the plant matrix. Plant cell walls are mainly composed of various structural polysaccharides such as cellulose, hemicellulose and pectin, as well as lignin and, in some cases, proteins [54]. Therefore, degrading pectin within the plant matrix does not necessarily result in a complete breakdown of the cell wall.

In contrast, Shahram et al. (2018) observed an increase in the extraction yield of phenolic compounds from orange processing waste when these compounds were extracted by a combined treatment of ultrasound and pectinase treatment. The suggested mechanism was either an increase in pectinase activity due to a change in pectinase conformation induced by ultrasound, or an increased permeability of the plant tissue due to a higher level of cell wall breakage by ultrasound [55]. Similarly, UAEE was found to yield the best results when compared to other green techniques to extract total phenolic compounds from apple pomace. The suggested mechanism involved improved solvent diffusion due to the particle size reduction, as well as enhanced mass transfer resulting from ultrasound-induced turbulence and cavitation [56].

## 4 Conclusion

In this study, the impact of US treatment in different modes (pre-, post-, and simultaneous treatment) on the susceptibility of different pectic substrates to enzymatic degradation was investigated. It was established that ultrasound did not enhance enzymatic degradation in any of the studied modes when applied to purified pectic substrates. However, in the case of pectin-rich biomass, ultrasound significantly improved enzymatic degradation when applied in the simultaneous mode (i.e., during enzymatic hydrolysis).

These results indicate that the effect of US treatment on the susceptibility of pectic substrates to enzymatic degradation is strongly dependent on the complexity of the biomass matrix. They suggest that ultrasound alone is insufficient to disrupt intact cell walls. However, when applied simultaneously with the enzyme(s), it increases the release of some metal ions from the biomass that, in turn, act as cofactors to improve the enzymatic degradation of pectin. It was also suggested that enzymatic pre-action may weaken the cell wall structure sufficiently to allow ultrasound-induced cavitation to further disrupt the matrix. On the other hand, results from the total phenolic content (TPC) analysis showed that despite the enhanced cell wall disruption observed under simultaneous ultrasound-enzyme treatment, none of the studied treatments resulted in a significant increase in total phenolic content compared to the non-degraded control.

## CRediT authorship contribution statement

**Bashar Kabawa:** Writing – review & editing, Writing – original draft, Visualization, Methodology, Investigation, Formal analysis, Conceptualization. **Imca Sampers:** Writing – review & editing, Supervision. **Katleen Raes:** Writing – review & editing, Supervision, Resources, Methodology, Funding acquisition, Conceptualization.

## Declaration of competing interest

The authors declare that they have no known competing financial interests or personal relationships that could have appeared to influence the work reported in this paper.
